# Association between metabolic syndrome and incident stroke: insights from the UK Biobank

**DOI:** 10.3389/fneur.2026.1803011

**Published:** 2026-04-13

**Authors:** Yan Zhou, Jicun Zhu, HuiMin Liu, Qiang Zhang, Jiajun Chen

**Affiliations:** 1 Department of Radiology, The First Affiliated Hospital of Zhengzhou University, Zhengzhou, Henan, China; 2 The First Affiliated Hospital of Zhengzhou University, Zhengzhou, Henan, China; 3 Department of Neurology, The First Affiliated Hospital of Zhengzhou University, Zhengzhou, Henan, China; 4 School of Nursing and Health, Zhengzhou University, Zhengzhou, Henan, China; 5 Department of Medical Services, The First Affiliated Hospital of Zhengzhou University, Zhengzhou, Henan, China

**Keywords:** association, hemorrhagic stroke, ischemic stroke, metabolic syndrome, stroke

## Abstract

**Background:**

Metabolic syndrome (MetS) is a cluster of metabolic abnormalities associated with increased cardiovascular risk. This study examined the association between MetS and incident stroke in the UK Biobank.

**Methods:**

This population-based prospective cohort study included 329,887 participants free of stroke at baseline. MetS was defined as the presence of at least three of the following components: elevated waist circumference, elevated blood glucose, elevated blood pressure, elevated triglycerides, and reduced high-density lipoprotein cholesterol. Incident all-cause stroke was identified using hospital inpatient records, death registries, and self-reported data, whereas ischemic and hemorrhagic stroke were identified using hospital records and death registries only. Cox proportional hazards models were used to evaluate the associations of MetS, its individual components, and the number of MetS components with incident stroke.

**Results:**

Over a median follow-up of 14.1 years, 6,716 incident stroke cases were recorded. MetS was associated with a higher risk of incident all-cause stroke (HR, 1.35; 95% CI, 1.28–1.42) and ischemic stroke (HR, 1.50; 95% CI, 1.41–1.60), but not hemorrhagic stroke (HR, 1.07; 95% CI, 0.96–1.20). Stroke risk increased with the number of MetS components. All individual MetS components were significantly associated with incident all-cause stroke, with elevated blood pressure showing the strongest association.

**Conclusion:**

MetS was significantly associated with incident all-cause stroke and ischemic stroke, but not hemorrhagic stroke, in this UK population.

## 1 Introduction

Stroke remains a critical public health issue, ranking as the third leading cause of death worldwide according to the Global Burden of Disease Study 2021 (1). In 2019, there were 12.2 million stroke cases and 6.55 million stroke-related deaths (2). Despite significant advances in emergency stroke treatment since 2015, the burden of the disease persists substantially. Given that approximately 85% of all strokes are preventable, emphasis on stroke prevention has gained traction (3). Modifiable lifestyle risk factors such as tobacco use, alcohol consumption, and cholesterol levels have gained increasing attention in stroke prevention. In Oxfordshire, stroke incidence fell by 32% from the Oxfordshire Community Stroke Project (1981–1986) to the Oxford Vascular Study (2002–2017), with a consistent downward trend before and after 2000 (4). Incorporating multiple population-based studies reported a 28% reduction in stroke incidence over an average period of 16.5 years between the 1990s and 2010s (4). These findings highlight the ongoing importance of identifying and addressing modifiable risk factors to further reduce stroke incidence.

Metabolic syndrome (MetS) is characterized by the presence of any three of five traits: abdominal obesity, elevated triglycerides, reduced high-density lipoprotein (HDL) cholesterol, prehypertension or hypertension, and insulin resistance or diabetes (5). MetS has become increasingly prevalent worldwide, with an estimated global prevalence of 21% among military personnel (6). Numerous studies and meta-analyses have established a link between MetS and an elevated risk of stroke (7–9). For instance, a meta-analysis of 16 studies involving 116,496 participants free of cardiovascular disease initially showed that individuals with MetS had a higher risk of stroke compared to those without MetS. This effect was more pronounced in females than in males, and was higher for ischemic stroke than hemorrhagic stroke (8). Additionally, combined analysis from the Atherosclerosis Risk in Communities Study and Jackson Heart Study indicated a consistent increase in ischemic stroke risk with MetS severity, particularly among White females, although interactions by sex and race were not significant (8). Further, a recent meta-analysis of 13 cohort studies comprising 59,919 participants over 60 years of age found a significant association between MetS and stroke recurrence (9).

Despite substantial evidence supporting the association between MetS and stroke risk, certain controversies and uncertainties remain. The strength of this relationship may vary across different ethnicities and geographic regions (10, 11). Additionally, the duration of follow-up can influence study outcomes, as shorter follow-up periods may underestimate the long-term impact of MetS on stroke risk (12). As MetS encompasses multiple metabolic abnormalities, its association with stroke risk may be further influenced by lifestyle factors such as smoking, alcohol consumption, and physical activity, complicating causal inference (13).

Moreover, evidence specifically focusing on the UK population remains limited. A UK cohort study involving 5,128 middle-aged men suggested an association between MetS and stroke risk (14); however, its findings were constrained by a relatively small sample size, the exclusion of women and older individuals, and a restricted demographic scope. To address these limitations, our study leverages the large-scale prospective UK Biobank cohort to provide a comprehensive analysis of the association between MetS and incident stroke risk, aiming to generate robust evidence for stroke prevention and management.

## 2 Methods

### 2.1 Study population

This study is presented according to the Strengthening the Reporting of Observational studies in Epidemiology guidelines (Supplementary Table S1) (15). The UK Biobank is a large population-based prospective cohort study of more than 500,000 participants recruited between 2006 and 2010 across the UK (16). Participants attended baseline assessment centers in England, Scotland, and Wales, where they provided electronically signed consent. At baseline, participants provided information on sociodemographic, lifestyle, environmental, and health-related factors through a touch-screen questionnaire and a nurse-led verbal interview (16, 17). UK Biobank received ethical approval from the National Health Service North West Centre for Research Ethics Committee.

**Figure 1:**
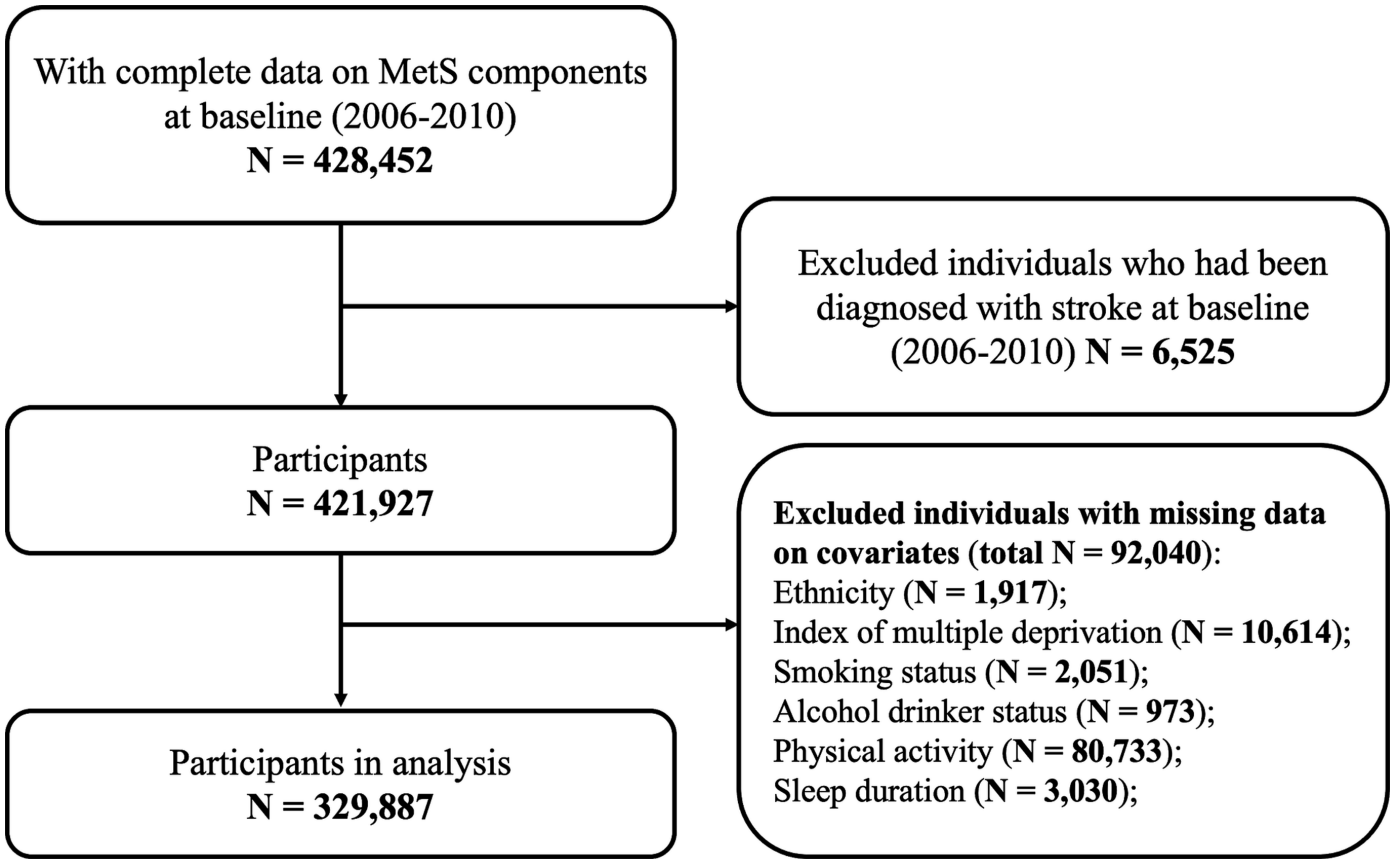
Flowchart of the study sample. MetS: metabolic syndrome.

In this present study, we included a sample of 428,452 individuals with complete data on all MetS components. We excluded 6,525 individuals who had been diagnosed with stroke prior to enrollment, and 92,040 individuals with missing data on covariates. Finally, 329,887 participants with complete data on all data were included in the main analysis. The flow chart of subject selection is shown in Figure 1.

### 2.2 Metabolic syndrome

In present study, MetS was determined according to the Harmonized Criteria from the International Diabetes Federation and the American Heart Association/National Heart, Lung, and Blood Institute established in 2009 (5). A diagnosis of MetS required the presence of at least three of the following five criteria: (1) elevated waist circumference (White, Black, and Mixed: waist circumference ≥102 cm for males and ≥88 cm for females; Asian: waist circumference ≥90 cm for males and ≥80 cm for females); (2) elevated fasting glucose (≥100 mg/dL or ≥5.6 mmol/L), and/or drug treatment for elevated glucose level, and/or diabetes diagnosed by doctor; Given that glucose levels in our study were primarily obtained from non-fasting blood samples, which can be highly variable due to recent food intake, we utilized glycated hemoglobin (HbA1c) as a proxy for glucose levels (18). Following the American Diabetes Association’s recommendations, we set a threshold of HbA1c ≥ 5.7% (39 mmol/mol) to indicate hyperglycemia (19). (3) elevated blood pressure (systolic ≥130 mmHg and/or diastolic ≥85 mmHg), and/or antihypertensive medication use, and/or high blood pressure diagnosed by doctor; (4) elevated triglycerides (≥150 mg/dL or 1.7 mmol/L) without cholesterol lowering medication use; and (5) reduced HDL-cholesterol (<40 mg/dL or 1.0 mmol/L in males; <50 mg/dL or 1.3 mmol/L in females).

Participants were divided into two groups: (1) those without MetS (reference group) and (2) those with MetS. Complete information regarding the variables and medication codes used to capture and define MetS components are available in Supplementary Table S2.

### 2.3 Stroke assessment

In the present study, incident stroke cases in the UK Biobank were identified using linked data from hospital inpatient records, death registries, and self-reported information. Stroke and its subtypes were ascertained using ICD-9 codes, ICD-10 codes, or self-reported data. When multiple stroke records were available for the same participant, the earliest recorded date of stroke occurrence was used, and the corresponding ascertainment source for that earliest event was considered the final source of diagnosis. Specifically, all-cause stroke was defined using ICD-9 codes 430, 431, 434, and 436, ICD-10 codes I60, I61, I63, and I64, and self-reported codes 1,081, 1,086, 1,491, and 1,583 (Field 20,002). Ischemic stroke was identified using ICD-9 codes 434 and 436 and ICD-10 code I63. Hemorrhagic stroke was identified using ICD-9 codes 430 and 431 and ICD-10 codes I60 and I61.

To reduce potential misclassification in subtype analyses, self-reported stroke cases and ICD-10 code I64 (unspecified stroke) were excluded from the analyses of ischemic and hemorrhagic stroke. The codes used to identify stroke outcomes in the UK Biobank are presented in Supplementary Table S3. Although individual neuroimaging data were not directly available in the UK Biobank, neuroimaging (CT/MRI) is a routine requirement for stroke diagnosis in hospital settings in the United Kingdom. Therefore, linkage to National Health Service (NHS) records provides relatively high diagnostic validity for stroke ascertainment (16).

### 2.4 Covariates

Covariates included potential confounders in the relationship between MetS and stroke (20). These covariates comprised sociodemographic factors (age, sex, ethnicity, and index of multiple deprivation), and lifestyle factors (smoking status, alcohol drinker status, physical activity, and sleep duration). In the main analysis, age was included as a continuous variable, whereas in the subgroup analysis, it was classified into two categories: ≤60 and >60. Sex was classified into female and male categories. Ethnicity was categorized as White, Mixed, Asian or Chinese, and Black. The index of multiple deprivation (IMD) is a comprehensive measure that encompasses various forms of deprivation experienced by individuals, including income (22.5%), employment (22.5%), health and disability (13.5%), education (13.5%), barriers to housing and services (9.3%), living environment (9.3%), and crime (9.3%) (21). The IMD was divided into two levels based on whether it was above or below the mean (IMD = 16.71). A higher IMD level was defined as >16.71, while a lower IMD level was defined as ≤16.71. Smoking status and alcohol drinker status were categorized as never, previous, and current. Physical activity levels were classified according to the International Physical Activity Questionnaire guidelines into low, moderate, and high (22). Sleep duration was categorized into short (<7 h per night), normal (7 h per night), and long (>7 h per night) (23). The field ID used in analyses is shown in Supplementary Table S4.

### 2.5 Statistical analyses

Descriptive statistics were utilized to compare baseline characteristics between participants with and without MetS. Continuous variables were presented as means with standard deviations (SD) for normally distributed data, and medians with interquartile ranges (IQR) for non-normally distributed data. Categorical variables were reported as numbers and percentages.

Multivariable Cox proportional-hazards models, using follow-up time as the underlying time scale, were employed to estimate the association of MetS, the number of MetS components (ranging from 0 to 5), and individual MetS components (elevated waist circumference, elevated HbA1c, elevated blood pressure, elevated triglycerides, and reduced HDL cholesterol) with incident all-cause stroke and its subtypes (ischemic and hemorrhagic stroke) (24). The Cox proportional hazards assumption was assessed using Schoenfeld residuals, and no significant violations were detected. Then, the cumulative incidence of stroke risk of MetS components (ranging from 0 to 5) was assessed using Kaplan–Meier curves. Differences between survival curves were evaluated using the log-rank test (25). Follow-up time (in years) for each participant began on the date of assessment center attendance and continued until the earliest occurrence of stroke diagnosis, loss to follow-up, death, or censoring on April 20, 2022. In present study, two models were used: the base model adjusted for sociodemographic factors (age, age^2^, sex, ethnicity, and index of multiple deprivation), and the final model adjusted for both socio-demographic factors, and lifestyle factors (smoking status, alcohol drinker status, physical activity, and sleep duration). Quadratic age terms (age^2^) were included to account for nonlinear age effects across all outcomes. Notably, the final model was considered to be the primary result. Results were presented as hazard ratios (HR) with 95% confidence intervals (CIs).

Subgroup analyses were performed to evaluate whether the association between MetS and incident all-cause stroke varied according to follow-up duration (≤5 years, >5 to 10 years, and >10 years), age (≤60 vs. >60 years), sex (female vs. male), IMD (lower vs. higher), smoking status (never, previous, current), alcohol drinking status (never, previous, current), physical activity (low, moderate, high), and sleep duration (<7 h, =7 h, and >7 h). *p* values for interaction were calculated to formally test potential effect modification across subgroups.

Several sensitivity analyses were performed to test the robustness and reliability of the association between MetS and the risk of incident all-cause stroke, including: (1) excluding participants with follow-up time less than 3 years to prevent potential bias; (2) removing participants with cardiovascular disease (heart attack and angina); (3) excluding cases with self-reported sources, retaining only stroke events identified through hospital inpatient records and death registries; (4) excluding patients with nontraumatic subarachnoid hemorrhagic; (5) re-analyzing within the White population due to the high proportion of White participants; and (6) using multiple imputation to assess the impact of missing data. Missing values for covariates (as detailed in Figure 1) were handled using multiple imputation by chained equations (MICE). Twenty imputed datasets were generated, and the analyses were repeated after imputation.

All *p* values were two-sided, with statistical significance set at *p* < 0.05. Analyses were performed using R, version 4.3.3.

## 3 Results

### 3.1 Population characteristics

The study included 329,887 participants with a mean (SD) age at recruitment of 56.3 (8.1) years. Participants with MetS were more likely to be older, male, Asian or Chinese, have higher IMD scores, be current or former smokers, never or former drinkers, less physically active, and have shorter or longer sleep durations compared to those without MetS. Among participants with MetS, 61.2% had three components, 29.9% had four, and 8.9% had five components. The most common MetS component was elevated blood pressure (90.8%), followed by elevated triglycerides (82.4%), larger waist circumference (78.9%), reduced HDL cholesterol (50.9%), and elevated HbA1c (44.7%). Over a median follow-up time of 14.1 years (IQR: 13.2–14.8 years), there were 6,716 cases of incident all-cause stroke, including 5,346 ischemic strokes and 1,661 hemorrhagic strokes. Baseline characteristics are detailed in Table 1. Participants characteristics according to incident all-cause stroke status at follow up are presented in Supplementary Table S5.

**Table 1:** Study cohort characteristics by MetS status.

Characteristic	Overall (*n* = 329,887)	No MetS (*n* = 240,512)	MetS (*n* = 89,375)
Person year, median (IQR), years	14.1 (13.2, 14.8)	14 (13.1, 14.7)	14.1 (13.3, 14.8)
Age, mean (SD), years	56.3 (8.1)	55.7 (8.2)	57.9 (7.7)
Age category, *n* (%)			
≤60	206,966 (62.7)	157,873 (65.6)	49,093 (54.9)
>60	122,921 (37.3)	82,639 (34.4)	40,282 (45.1)
Sex, *n* (%)			
Female	171,568 (52.0)	129,057 (53.7)	42,511 (47.6)
Male	158,319 (48.0)	111,455 (46.3)	46,864 (52.4)
Ethnicity, *n* (%)			
White	313,689 (95.1)	230,539 (95.9)	83,150 (93.1)
Black	4,699 (1.4)	3,254 (1.4)	1,445 (1.6)
Asian or Chinese	6,892 (2.1)	3,397 (1.4)	3,495 (3.9)
Mixed	4,607 (1.4)	3,322 (1.3)	1,285 (1.4)
IMD, *n* (%)			
Low	210,574 (63.8)	158,797 (66.0)	51,777 (57.9)
High	119,313 (36.2)	81,715 (34.0)	37,598 (42.1)
Smoking status, *n* (%)			
Never	181,141 (54.9)	136,943 (56.9)	44,198 (49.5)
Previous	115,378 (35.0)	80,728 (33.6)	34,650 (38.8)
Current	33,368 (10.1)	22,841 (9.5)	10,527 (11.8)
Alcohol drinker status, *n* (%)			
Never	12,910 (3.9)	7,730 (3.2)	5,180 (5.8)
Previous	11,112 (3.4)	6,904 (2.9)	4,208 (4.7)
Current	305,865 (92.7)	225,878 (93.9)	79,987 (89.5)
Physical activity, *n* (%)			
Low	61,807 (18.7)	39,326 (16.4)	22,481 (25.2)
Moderate	134,545 (40.8)	97,471 (40.5)	37,074 (41.5)
High	133,535 (40.5)	103,715 (43.1)	29,820 (33.4)
Sleep duration, *n* (%)			
Short (<7 h per night)	79,044 (24.0)	55,372 (23.0)	23,672 (26.5)
Normal (7 h per night)	130,137 (39.4)	98,387 (40.9)	31,750 (35.5)
Long (>7 h per night)	120,706 (36.6)	86,753 (36.1)	33,953 (38.0)
Larger waist circumference, *n* (%)	108,797 (33.0)	38,247 (15.9)	70,550 (78.9)
Elevated triglyceride levels, *n* (%)	131,283 (39.8)	57,682 (24.0)	73,601 (82.4)
Elevated blood pressure, *n* (%)	220,459 (66.8)	139,332 (57.9)	81,127 (90.8)
Elevated HbA1c, *n* (%)	56,871 (17.2)	16,902 (7.0)	39,969 (44.7)
Reduced HDL cholesterol level, *n* (%)	63,599 (19.3)	18,118 (7.5)	45,481 (50.9)
All-cause stroke, *n* (%)	6,716 (2.0)	4,145 (1.7)	2,571 (2.9)
Ischemic stroke, *n* (%)	5,346 (1.6)	3,191 (1.3)	2,155 (2.4)
Hemorrhagic stroke, *n* (%)	1,661 (0.5)	1,127 (0.5)	534 (0.6)

MetS: metabolic syndrome; IQR: interquartile range; SD: standard deviation; IMD: index of multiple deprivation; HbA1c: glycosylated hemoglobin A1c; HDL: high-density lipoprotein.

### 3.2 Association between MetS and stroke

**Figure 2:**
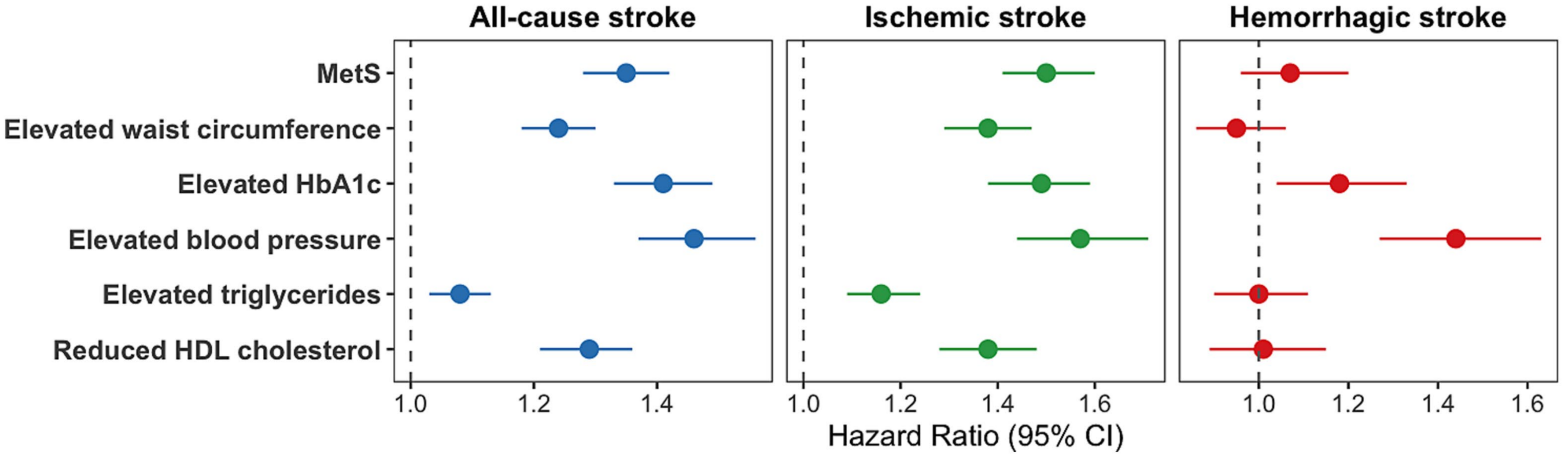
Association between MetS, MetS components, and the risk of stroke. Adjusted for age, age^2^, sex, ethnicity, and index of multiple deprivation, smoking status, alcohol drinker status, physical activity, and sleep duration. MetS: metabolic syndrome; CI: confidence interval.

Compared to participants without MetS, those with MetS had a higher risk of incident all-cause stroke (final adjusted HR: 1.35, 95% CI: 1.28–1.42, Figure 2; Supplementary Table S6) and ischemic stroke (final adjusted HR: 1.50, 95% CI: 1.41–1.60, Figure 2; Supplementary Table S7). While, there was no significant association between MetS and hemorrhagic stroke (final adjusted HR: 1.07, 95% CI: 0.96–1.20, Figure 2; Supplementary Table S8).

**Figure 3:**
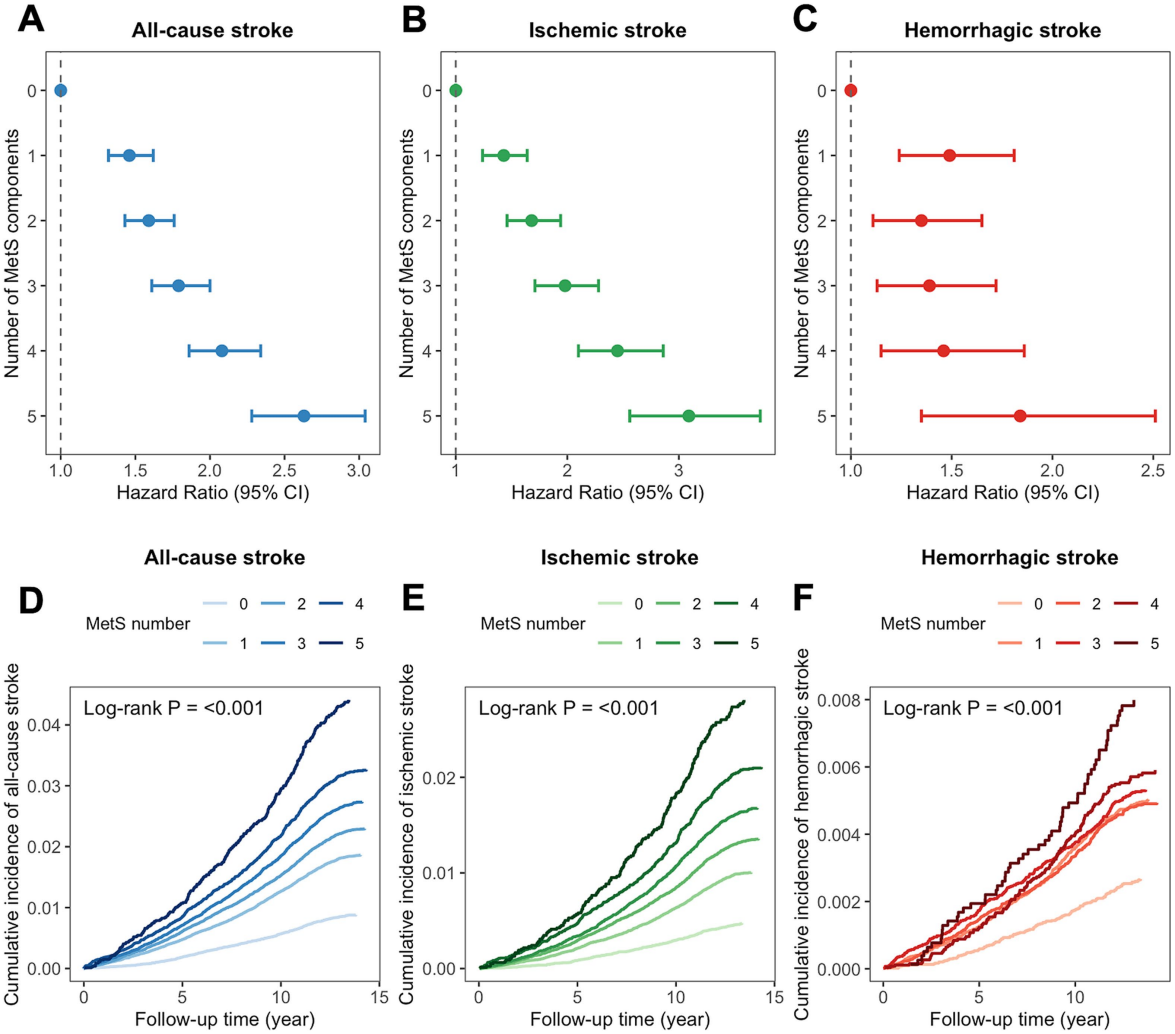
Relationship between the number of MetS components and the incidence of stroke. Panels **(A–C)** display the Cox proportional-hazards model examining the association between the number of MetS components and the incidence of all-cause stroke, ischemic stroke, and hemorrhagic stroke, respectively. Panels **(D–F)** present Kaplan–Meier curves illustrating the cumulative incidence of all-cause stroke, ischemic stroke, and hemorrhagic stroke according to the number of MetS components. Adjusted for age, age^2^, sex, ethnicity, and index of multiple deprivation, smoking status, alcohol drinker status, physical activity, and sleep duration. MetS: metabolic syndrome; CI: confidence interval.

The risk of incident all-cause stroke, ischemic stroke, and hemorrhagic stroke increased with the number of MetS components (from 1 to 5) (Figures 3A–C; Supplementary Tables S6–S8, *p* values < 0.001). The risk of incident stroke showed an incremental trend with more components present. Kaplan–Meier curves confirmed these findings (log-rank test, *p* values < 0.001, Figures 3D–F).

Each MetS component, including elevated waist circumference, elevated HbA1c, elevated blood pressure, elevated triglycerides, and reduced HDL cholesterol, was linked to an increased risk of incident all-cause stroke and ischemic stroke (Figure 2; Supplementary Tables S6, S7). Elevated HbA1c and elevated blood pressure were also associated with a higher risk of incident hemorrhagic stroke. However, larger waist circumference, reduced HDL cholesterol, and elevated triglycerides were not significantly associated with incident hemorrhagic stroke (Figure 2; Supplementary Table S8). Furthermore, we found that compared to other individual MetS components, elevated blood pressure showed the highest risk of incident all-cause stroke (final adjusted HR: 1.46, 95% CI: 1.37–1.56), ischemic stroke (final adjusted HR: 1.57, 95% CI: 1.44–1.71), and hemorrhagic stroke (final adjusted HR: 1.44, 95% CI: 1.27–1.63).

### 3.3 Additional analyses

In subgroup analyses, there was no significant interaction between MetS and age, sex, IMD, smoking status, alcohol drinking status, physical activity, or sleep duration in relation to incident all-cause stroke (all *p* for interaction > 0.05, Table 2). In contrast, a significant interaction was observed for follow-up duration (*p* for interaction < 0.001). In the fully adjusted model, the association between MetS and incident all-cause stroke was not statistically significant among participants with ≤5 years of follow-up (HR, 1.05; 95% CI, 0.95–1.16) or 5–10 years of follow-up (HR, 1.03; 95% CI, 0.96–1.12), but remained significant among those with >10 years of follow-up (HR, 1.36; 95% CI, 1.24–1.49) (Table 2).

**Table 2:** Subgroup analyses of association between MetS and all-cause stroke.

Variables	Subgroup	Base model			Final model		
		HR (95% CI)	*p*	*p* for interaction	HR (95% CI)	*p*	*p* for interaction
Follow-up time (year)	≤5	1.04 (0.95–1.15)	0.41	<0.001	1.05 (0.95–1.16)	0.306	<0.001
	5 ~ 10	1.01 (0.94–1.09)	0.742		1.03 (0.96–1.12)	0.404	
	>10	1.41 (1.29–1.55)	<0.001		1.36 (1.24–1.49)	<0.001	
Age	≤60	1.60 (1.47–1.74)	<0.001	0.09	1.55 (1.42–1.68)	<0.001	0.063
	>60	1.30 (1.23–1.39)	<0.001		1.26 (1.18–1.34)	<0.001	
Sex	Female	1.46 (1.35–1.58)	<0.001	0.123	1.39 (1.29–1.51)	<0.001	0.09
	Male	1.36 (1.28–1.45)	<0.001		1.32 (1.23–1.40)	<0.001	
IMD	Lower	1.37 (1.28–1.46)	<0.001	0.384	1.34 (1.25–1.43)	<0.001	0.387
	Higher	1.45 (1.34–1.56)	<0.001		1.37 (1.27–1.48)	<0.001	
Smoking status	Never	1.43 (1.33–1.54)	<0.001	0.421	1.43 (1.32–1.54)	<0.001	0.494
	Previous	1.33 (1.23–1.43)	<0.001		1.30 (1.20–1.40)	<0.001	
	Current	1.36 (1.20–1.54)	<0.001		1.31 (1.15–1.49)	<0.001	
Alcohol drinker status	Never	1.28 (1.03–1.60)	0.026	0.299	1.21 (0.97–1.51)	0.087	0.278
	Previous	1.59 (1.28–1.97)	<0.001		1.53 (1.22–1.90)	<0.001	
	Current	1.38 (1.31–1.46)	<0.001		1.35 (1.28–1.42)	<0.001	
Physical activity	Low	1.41 (1.27–1.57)	<0.001	0.667	1.35 (1.21–1.51)	<0.001	0.695
	Moderate	1.35 (1.24–1.46)	<0.001		1.30 (1.20–1.41)	<0.001	
	High	1.42 (1.31–1.54)	<0.001		1.39 (1.29–1.51)	<0.001	
Sleep duration	<7 h	1.34 (1.22–1.48)	<0.001	0.593	1.30 (1.17–1.43)	<0.001	0.569
	=7 h	1.44 (1.32–1.57)	<0.001		1.40 (1.28–1.53)	<0.001	
	>7 h	1.40 (1.29–1.51)	<0.001		1.33 (1.23–1.44)	<0.001	

The base model adjusted for sociodemographic factors (age, age^2^, sex, ethnicity, and index of multiple deprivation), and the final model adjusted for both socio-demographic factors, and lifestyle factors (smoking status, alcohol drinker status, physical activity, and sleep duration). HR: hazard ratio; CI: confidence interval; IMD: index of multiple deprivation; HbA1c: glycosylated hemoglobin A1c; HDL: high-density lipoprotein; MetS: metabolic syndrome.

Sensitivity analyses demonstrated that the associations remained robust after (1) excluding participants with a follow-up time of less than 3 years (*N* = 290,086); (2) excluding participants with pre-existing cardiovascular disease (*N* = 315,096); (3) excluding stroke cases identified through self-reported sources; (4) excluding participants with nontraumatic subarachnoid hemorrhage (*N* = 329,218); (5) restricting the analysis to White participants (*N* = 313,689); and (6) applying multiple imputation to account for missing covariate data (Complete-case *N* = 329,887; Multiple imputation *N* = 421,927) (all *p* < 0.05, Supplementary Table S9).

## 4 Discussion

This population-based cohort study of 329,887 individuals in the UK demonstrates a significant association between MetS and an elevated risk of incident all-cause stroke. With a 35% increased risk of incident all-cause stroke observed over a 14-year follow-up period. This finding remained robust across multiple sensitivity analyses, underscoring the consistency and reliability of our results. Notably, the risk of incident stroke exhibited a dose–response relationship with the number of MetS components present. Among the various components of MetS, elevated blood pressure emerged as the most influential factor contributing to the risk of incident stroke.

Our study reveals a significant association between MetS and an elevated risk of all-cause stroke. These results align with the majority of previous observational studies conducted in other regions, such as Asia (e.g., Japan and China) (26–33) and the United States (34–36), which have consistently identified MetS as a significant risk factor for stroke. One notable cohort study conducted in the UK involving 5,128 men aged 40 to 59 years without a history of cardiovascular disease or type 2 diabetes found that men with MetS at baseline exhibited a significantly higher relative risk of developing stroke compared to those without MetS (14). This evidence, albeit limited by age and gender, underscores the heightened stroke risk associated with MetS in a specific segment of the British population. Our research expands upon these findings by incorporating a more extensive and diverse population. Through subgroup analysis by gender, we observed that men with MetS at baseline had a significantly higher risk of developing stroke (HR, 1.32; 95% CI, 1.23–1.40) compared to those without MetS. This robust association supports and extends the earlier observations by providing a more comprehensive understanding of the impact of MetS on stroke risk across a broader age range and inclusive of males and females.

In addition to observational evidence, Mendelian randomization studies have provided genetic support for a potential causal relationship between MetS and stroke. For example, one Mendelian randomization study reported that genetically predicted MetS was associated with a 1.05-fold higher risk of stroke (95% CI: 1.02–1.09, *p* = 0.0012) (37). Our findings are directionally consistent with this evidence, as we also observed that MetS was associated with an increased risk of incident stroke in this large prospective cohort. However, these two lines of evidence are not directly equivalent: Mendelian randomization reflects lifelong genetic predisposition, whereas our study assessed clinically defined baseline MetS in a real-world population. Therefore, differences in effect estimates may reflect differences in exposure definition, residual confounding, and changes in metabolic status or treatment over time.

Our study found that the association between MetS and the risk of incident stroke varies by stroke subtype. Compared to participants without MetS, those with MetS had a higher risk of ischemic stroke, while no significant association was observed between MetS and hemorrhagic stroke. These findings are corroborated by two cohort studies from Japan (30, 32). One study indicated that MetS is an independent risk factor for hemorrhagic stroke only in men (30). Another study, involving 2,613 subjects aged 40–69 from a rural Japanese community, reported multivariable HR (95% CI) associated with MetS as 1.7 (1.0–2.7) for total stroke, 2.0 (1.2–3.5) for ischemic stroke, and 1.1 (0.4–2.8) for hemorrhagic stroke (32). Similarly, a study conducted among Chinese individuals aged 35 to 64 showed that MetS was associated with a higher risk of both ischemic stroke and hemorrhagic stroke (27). A meta-analysis of these studies further demonstrated that MetS is more strongly associated with ischemic stroke (HR = 2.12, 95% CI: 1.46–3.08) than hemorrhagic stroke (HR = 1.48, 95% CI: 0.98–2.24), which is in complete agreement with our findings (7). This disparity may be due to MetS being closely linked to atherosclerosis, a major cause of ischemic stroke (38). Components of MetS, such as hypertension, hyperglycemia, dyslipidemia, and abdominal obesity, contribute to the formation of atherosclerotic plaques and thrombus formation, which can block cerebral arteries and lead to ischemic stroke. This pathophysiological mechanism underscores the heightened risk of ischemic stroke in individuals with MetS and highlights the need for targeted interventions addressing these specific metabolic components to mitigate the overall stroke risk (38).

Additionally, this present study found that compared to other individual MetS components, elevated blood pressure showed the highest risk of all-cause stroke, ischemic stroke, and hemorrhagic stroke. The impact of elevated blood pressure on stroke incidence has been examined across various countries, including the Netherlands (39), China (25, 40), and the USA (41, 42). Our findings are consistent with this existing research and add new evidence from the UK population. There is often a delay between the onset of hypertension and the development of hypertensive complications. During this prolonged period, a series of changes occur within the cardiovascular system, including the cerebral circulation. These changes, such as vascular remodeling, inflammation, oxidative stress, and baroreflex dysfunction, may contribute to the pathogenesis of stroke in hypertensive individuals (43). This emphasizes the critical importance of early detection and management of elevated blood pressure to mitigate the long-term risk of stroke.

A significant interaction was observed by follow-up duration, with the association between MetS and incident stroke becoming apparent only after more than 10 years of follow-up. This pattern should be interpreted cautiously. It may reflect time-dependent effects of MetS, differences in early-event dynamics, or exposure misclassification over time, as MetS status was assessed only at baseline and may have changed during follow-up. Future studies using repeated metabolic assessments and time-varying exposure models are needed to clarify this finding.

Our study has several strengths. First, it is based on a large, population-based cohort of 329,887 individuals, which enhances the generalizability of our findings to the broader UK population. The extensive follow-up period of 14 years provides robust data on the long-term association between MetS and the risk of incident stroke. Additionally, the comprehensive collection of baseline data, including sociodemographic, lifestyle, and health-related factors, allows for detailed adjustment of potential confounders, thereby strengthening the validity of our results. Furthermore, the inclusion of a wide range of sensitivity analyses, such as excluding participants with pre-existing cardiovascular disease and using multiple imputation for missing data, supports the reliability of our findings.

Despite the robustness of our findings, several limitations should be noted. First, the observational design precludes causal inference. Second, because blood samples were primarily collected under non-fasting conditions, HbA1c was used as a proxy for hyperglycemia; however, HbA1c is not directly equivalent to fasting glucose in the harmonized MetS criteria, which may affect comparability with previous studies. Third, stroke outcomes were primarily identified from hospital records and death registries using ICD codes. Although neuroimaging is routinely required for stroke diagnosis in clinical practice, individual imaging data were unavailable, and some outcome misclassification may have been unavoidable. TOAST-based etiologic classification was also unavailable, limiting further analyses of ischemic stroke subtypes. Fourth, although sensitivity analyses excluding self-reported stroke cases yielded consistent results, recall bias related to self-reported data cannot be completely excluded. Fifth, because MetS was defined using baseline measurements only, changes in metabolic status over time may have led to exposure misclassification and attenuation of the observed associations. Sixth, the predominance of participants of European ancestry may limit the generalizability of our findings. Finally, several covariates were self-reported at baseline and may therefore be subject to reporting bias.

## 5 Conclusion

In conclusion, this large population-based cohort study showed that MetS was significantly associated with an increased risk of incident all-cause stroke and ischemic stroke, but not hemorrhagic stroke, in the UK population. Among the individual MetS components, elevated blood pressure showed the strongest association with incident stroke. These findings highlight the importance of early identification and management of MetS, particularly hypertension, for stroke prevention. Further studies are needed to clarify the underlying mechanisms and to inform targeted prevention strategies.

## Data Availability

The original contributions presented in the study are included in the article/Supplementary material, further inquiries can be directed to the corresponding author.
