# Supplementary material for: Association between metabolic syndrome and incident stroke: insights from the UK Biobank
Source: Front Neurol. 2026 Apr 13;17:1803011. doi: 10.3389/fneur.2026.1803011 (PMC13122770; doi:10.3389/fneur.2026.1803011)
Supplement: Supplementary file 1 [file Supplementary_file_1.docx]

**Table S1. STROBE Statement—Checklist of items that should be included in reports of *cohort studies***

|  | **Item No** | **Recommendation** | **Page No** |
| --- | --- | --- | --- |
| **Title and abstract** | 1 | (*a*) Indicate the study’s design with a commonly used term in the title or the abstract |  |
|  |  | (*b*) Provide in the abstract an informative and balanced summary of what was done and what was found |  |
| **Introduction** | | | |
| Background/rationale | 2 | Explain the scientific background and rationale for the investigation being reported |  |
| Objectives | 3 | State specific objectives, including any prespecified hypotheses |  |
| **Methods** | | | |
| Study design | 4 | Present key elements of study design early in the paper |  |
| Setting | 5 | Describe the setting, locations, and relevant dates, including periods of recruitment, exposure, follow-up, and data collection |  |
| Participants | 6 | (*a*) Give the eligibility criteria, and the sources and methods of selection of participants. Describe methods of follow-up |  |
|  |  | (*b*) For matched studies, give matching criteria and number of exposed and unexposed |  |
| Variables | 7 | Clearly define all outcomes, exposures, predictors, potential confounders, and effect modifiers. Give diagnostic criteria, if applicable |  |
| Data sources/ measurement | 8* | For each variable of interest, give sources of data and details of methods of assessment (measurement). Describe comparability of assessment methods if there is more than one group |  |
| Bias | 9 | Describe any efforts to address potential sources of bias |  |
| Study size | 10 | Explain how the study size was arrived at |  |
| Quantitative variables | 11 | Explain how quantitative variables were handled in the analyses. If applicable, describe which groupings were chosen and why |  |
| Statistical methods | 12 | (*a*) Describe all statistical methods, including those used to control for confounding |  |
|  |  | (*b*) Describe any methods used to examine subgroups and interactions |  |
|  |  | (*c*) Explain how missing data were addressed |  |
|  |  | (*d*) If applicable, explain how loss to follow-up was addressed |  |
|  |  | (*e*) Describe any sensitivity analyses |  |
| **Results** | | |  |
| Participants | 13* | (a) Report numbers of individuals at each stage of study—eg numbers potentially eligible, examined for eligibility, confirmed eligible, included in the study, completing follow-up, and analysed |  |
|  |  | (b) Give reasons for non-participation at each stage |  |
|  |  | (c) Consider use of a flow diagram |  |
| Descriptive data | 14* | (a) Give characteristics of study participants (eg demographic, clinical, social) and information on exposures and potential confounders |  |
|  |  | (b) Indicate number of participants with missing data for each variable of interest |  |
|  |  | (c) Summarise follow-up time (eg, average and total amount) |  |
| Outcome data | 15* | Report numbers of outcome events or summary measures over time |  |
| Main results | 16 | (a) Give unadjusted estimates and, if applicable, confounder-adjusted estimates and their precision (eg, 95% confidence interval). Make clear which confounders were adjusted for and why they were included |  |
|  |  | (b) Report category boundaries when continuous variables were categorized |  |
|  |  | (c) If relevant, consider translating estimates of relative risk into absolute risk for a meaningful time period |  |
| Other analyses | 17 | Report other analyses done—eg analyses of subgroups and interactions, and sensitivity analyses |  |
| **Discussion** | | | |
| Key results | 18 | Summarise key results with reference to study objectives |  |
| Limitations | 19 | Discuss limitations of the study, taking into account sources of potential bias or imprecision. Discuss both direction and magnitude of any potential bias |  |
| Interpretation | 20 | Give a cautious overall interpretation of results considering objectives, limitations, multiplicity of analyses, results from similar studies, and other relevant evidence |  |
| Generalisability | 21 | Discuss the generalisability (external validity) of the study results |  |
| **Other information** | | | |
| Funding | 22 | Give the source of funding and the role of the funders for the present study and, if applicable, for the original study on which the present article is based |  |

**Table S2. Variables codes used to define MetS in the UK Biobank cohort**

| Metabolic syndrome component | Harmonized criteria (2009)  *Three or more of the following:* | UKB data field ID | Field code description |
| --- | --- | --- | --- |
| Elevated waist circumference | Elevated waist circumference: ≥ 102 cm in males and ≥ 88 cm in females | 48 | Waist circumference |
| Elevated triglycerides | ≥ 150 mg/dL (1.7 mmol/L) | 30870 | Triglycerides Blood Biochemistry |
| Elevated blood pressure | Systolic: ≥130 mmHg and/or Diastolic ≥85 mmHg, or antihypertensive medication use, or high blood pressure diagnosed by doctor  In UKB, BP was measured twice by trained nurses after participants had been at rest for at least 5 minutes. Individual systolic and diastolic BP measurements were averaged within a visit. Automated BP readings were the preferred source of data. | 4079  4080  6153  6177  6150 | Diastolic BP - automated reading Systolic BP - automated reading Used high blood pressure medication  Used high blood pressure medication  High blood pressure diagnosed by doctor |
| Elevated blood glucose | ≥100 mg/dL (≥5.6 mmol/L), or drug treatment for elevated glucose level, or diabetes diagnosed by doctor  HbA1c used as a proxy indicator, with cut-offs based on the recommendations of the American Diabetes Association: HbA1c ≥5.7% | 30750  2443  6153  6177 | HbA1c  Diabetes diagnosed by doctor  Used insulin  Used insulin |
| Reduced HDL cholesterol | Males: <40 mg/dL (1 mmol/L); Females: <50 mg/dL (1.3 mmol/L) | 30760  20003 | HDL cholesterol  ATC Codes starting with: C10 |

*HDL = High-density lipoprotein, BP = Blood pressure, HbA1c = Glycated haemoglobin*

**Table S3. Codes used in the UK Biobank cohort to identify stroke cases**

| Cause | ICD-9 | ICD-10 | Self-reported UK Biobank field code |
| --- | --- | --- | --- |
| All cause stroke | 430; 431; 434; 436 | I60; I61; I63; I64 | Field 20002 Code 1081, 1086, 1491, 1583 |
| Ischemic stroke | 434; 436 | I63 | / |
| Hemorrhagic stroke | 430; 431 | I60; I61 | / |

**Table S4. Field ID of covariates used in the UK Biobank cohort.**

| Covariates | Field ID | Values |
| --- | --- | --- |
| Age | 21022 | Continuous |
| Sex | 31 | 1 “Male”; 2 “female” |
| Ethnicity | 21000 | 1 “White”; 2 “Mixed”; 3 “Asian or Chinese”; 4 “Black”; 5 “Others” |
| Index of Multiple Deprivation (England) | 26410 | 1 “Lower”; 2 “Higher” |
| Index of Multiple Deprivation (Scotland) | 26427 | 1 “Lower”; 2 “Higher” |
| Index of Multiple Deprivation (Wales) | 26426 | 1 “Lower”; 2 “Higher” |
| Smoking status | 20116 | 0 “Never”; 1 “Previous”; 2 “Current” |
| Alcohol drinker status | 20117 | 0 “Never”; 1 “Previous”; 2 “Current” |
| Physical activity | 22032 | 1 “Low”; 2 “Moderate”; 3 “High” |
| Sleep duration | 1160 | 1 “short (<7 hours per night)”; 2 “normal (7 hours per night)”; 3 “long (>7 hours per night)” |
| Attendance date, lost date, and death date | 53, 191, and 40000 | Month-Day-year |

**Table S5. Study cohort characteristics by incident stroke status.**

| Characteristic | Overall  (n = 329,887) | No stroke  (n = 323,171) | Stroke  (n = 6,716) |
| --- | --- | --- | --- |
| Person year, median (IQR), years | 14.1 (13.2, 14.8) | 14.1 (13.3, 14.8) | 8.0 (4.8, 10.6) |
| Age, mean (SD), years | 56.3 (8.1) | 56.2 (8.1) | 61.3 (6.7) |
| Age category, n (%) |  |  |  |
| ≤ 60 | 206,966 (62.7) | 204,567 (63.3) | 2,399 (35.7) |
| > 60 | 122,921 (37.3) | 118,604 (36.7) | 4,317 (64.3) |
| Sex, n (%) |  |  |  |
| Female | 171,568 (52.0) | 168,893 (52.3) | 2,675 (39.8) |
| Male | 158,319 (48.0) | 154,278 (47.7) | 4,041 (60.2) |
| Ethnicity, n (%) |  |  |  |
| White | 313,689 (95.1) | 307,261 (95.1) | 6,428 (95.8) |
| Black | 4,699 (1.4) | 4,598 (1.4) | 101 (1.5) |
| Asian or Chinese | 6,892 (2.1) | 6,762 (2.1) | 130 (1.9) |
| Mixed | 4,607 (1.4) | 4,550 (1.4) | 57 (0.8) |
| IMD, n (%) |  |  |  |
| Low | 210,574 (63.8) | 206,571 (63.9) | 4,003 (59.6) |
| High | 119,313 (36.2) | 116,600 (36.1) | 2,713 (40.4) |
| Smoking status, n (%) |  |  |  |
| Never | 181,141 (54.9) | 178,117 (55.1) | 3,024 (45.0) |
| Previous | 115,378 (35.0) | 112,682 (34.9) | 2,696 (40.1) |
| Current | 33,368 (10.1) | 32,372 (10.0) | 996 (14.8) |
| Alcohol drinker status, n (%) |  |  |  |
| Never | 12,910 (3.9) | 12,581 (3.9) | 329 (4.9) |
| Previous | 11,112 (3.4) | 10,784 (3.3) | 328 (4.9) |
| Current | 305,865 (92.7) | 299,806 (92.8) | 6,059 (90.2) |
| Physical activity, n (%) |  |  |  |
| Low | 61,807 (18.7) | 60,442 (18.7) | 1,365 (20.3) |
| Moderate | 134,545 (40.8) | 131,933 (40.8) | 2,612 (38.9) |
| High | 133,535 (40.5) | 130,796 (40.5) | 2,739 (40.8) |
| Sleep duration, n (%) |  |  |  |
| Short (<7 hours per night) | 79,044 (24.0) | 77,360 (23.9) | 1,684 (25.1) |
| Normal (7 hours per night) | 130,137 (39.4) | 127,833 (39.6) | 2,304 (34.3) |
| Long (>7 hours per night) | 120,706 (36.6) | 117,978 (36.5) | 2,728 (40.6) |
| Larger waist circumference, n (%) | 108,797 (33.0) | 106,085 (32.8) | 2,712 (40.4) |
| Elevated triglyceride levels, n (%) | 131,283 (39.8) | 128,193 (39.7) | 3,090 (46.0) |
| Elevated blood pressure, n (%) | 220,459 (66.8) | 215,007 (66.5) | 5,452 (81.2) |
| Elevated HbA1c, n (%) | 56,871 (17.2) | 54,947 (17.0) | 1,924 (28.6) |
| Reduced HDL cholesterol level, n (%) | 63,599 (19.3) | 62,059 (19.2) | 1,540 (22.9) |
| MetS, n (%) | 89,375 (27.1) | 86,804 (26.9) | 2,571 (38.3) |

IQR: interquartile range; SD: standard deviation; IMD: index of multiple deprivation; HbA1c: glycosylated hemoglobin A1c; HDL: high-density lipoprotein; MetS: Metabolic syndrome.

**Table S6. Association of MetS with incident all-cause stroke**

| Index | Base model | | Final model | |
| --- | --- | --- | --- | --- |
|  | HR (95% CI) | *P* | HR (95% CI) | *P* |
| MetS | 1.40 (1.33, 1.47) | <0.001 | 1.35 (1.28, 1.42) | <0.001 |
| Number of MetS components |  |  |  |  |
| 0 | 1 (1, 1) |  | 1 (1, 1) |  |
| 1 | 1.47 (1.32, 1.63) | <0.001 | 1.46 (1.32, 1.62) | <0.001 |
| 2 | 1.62 (1.46, 1.80) | <0.001 | 1.59 (1.43, 1.76) | <0.001 |
| 3 | 1.86 (1.67, 2.06) | <0.001 | 1.79 (1.61, 2.00) | <0.001 |
| 4 | 2.20 (1.96, 2.46) | <0.001 | 2.08 (1.86, 2.34) | <0.001 |
| 5 | 2.83 (2.46, 3.27) | <0.001 | 2.63 (2.28, 3.04) | <0.001 |
| Individual MetS components |  |  |  |  |
| Elevated waist circumference | 1.28 (1.22, 1.34) | <0.001 | 1.24 (1.18, 1.30) | <0.001 |
| Elevated HbA1c | 1.47 (1.39, 1.55) | <0.001 | 1.41 (1.33, 1.49) | <0.001 |
| Elevated blood pressure | 1.45 (1.36, 1.54) | <0.001 | 1.46 (1.37, 1.56) | <0.001 |
| Elevated triglycerides | 1.11 (1.06, 1.17) | <0.001 | 1.08 (1.03, 1.13) | <0.001 |
| Reduced HDL cholesterol | 1.34 (1.27, 1.42) | <0.001 | 1.29 (1.21, 1.36) | <0.001 |

The base model adjusted for sociodemographic factors (age, age^2^, sex, ethnicity, and index of multiple deprivation), and the final model adjusted for both socio-demographic factors, and lifestyle factors (smoking status, alcohol drinker status, physical activity, and sleep duration). HR: hazard ratio; CI: confidence interval; IMD: index of multiple deprivation; HbA1c: glycosylated hemoglobin A1c; HDL: high-density lipoprotein; MetS: Metabolic syndrome.

**Table S7. Association of MetS with incident ischemic stroke**

| Index | Base model | | Final model | |
| --- | --- | --- | --- | --- |
|  | HR (95% CI) | *P* | HR (95% CI) | *P* |
| MetS | 1.54 (1.45, 1.65) | <0.001 | 1.50 (1.41, 1.60) | <0.001 |
| Number of MetS components |  |  |  |  |
| 0 | 1 (1, 1) |  | 1 (1, 1) |  |
| 1 | 1.43 (1.24, 1.64) | <0.001 | 1.43 (1.24, 1.64) | <0.001 |
| 2 | 1.71 (1.48, 1.96) | <0.001 | 1.68 (1.46, 1.94) | <0.001 |
| 3 | 2.02 (1.75, 2.34) | <0.001 | 1.98 (1.71, 2.28) | <0.001 |
| 4 | 2.54 (2.18, 2.96) | <0.001 | 2.45 (2.10, 2.86) | <0.001 |
| 5 | 3.25 (2.69, 3.91) | <0.001 | 3.09 (2.56, 3.73) | <0.001 |
| Individual MetS components |  |  |  |  |
| Elevated waist circumference | 1.40 (1.32, 1.50) | <0.001 | 1.38 (1.29, 1.47) | <0.001 |
| Elevated HbA1c | 1.54 (1.44, 1.65) | <0.001 | 1.49 (1.38, 1.59) | <0.001 |
| Elevated blood pressure | 1.55 (1.43, 1.69) | <0.001 | 1.57 (1.44, 1.71) | <0.001 |
| Elevated triglycerides | 1.19 (1.12, 1.27) | <0.001 | 1.16 (1.09, 1.24) | <0.001 |
| Reduced HDL cholesterol | 1.42 (1.32, 1.53) | <0.001 | 1.38 (1.28, 1.48) | <0.001 |

The base model adjusted for sociodemographic factors (age, age^2^, sex, ethnicity, and index of multiple deprivation), and the final model adjusted for both socio-demographic factors, and lifestyle factors (smoking status, alcohol drinker status, physical activity, and sleep duration). HR: hazard ratio; CI: confidence interval; IMD: index of multiple deprivation; HbA1c: glycosylated hemoglobin A1c; HDL: high-density lipoprotein; MetS: Metabolic syndrome.

**Table S8. Association of MetS with incident hemorrhagic stroke**

| Index | Base model | | Final model | |
| --- | --- | --- | --- | --- |
|  | HR (95% CI) | *P* | HR (95% CI) | *P* |
| MetS | 1.11 (1.00, 1.24) | 0.061 | 1.07 (0.96, 1.20) | 0.205 |
| Number of MetS components |  |  |  |  |
| 0 | 1 (1, 1) |  | 1 (1, 1) |  |
| 1 | 1.50 (1.24, 1.81) | <0.001 | 1.49 (1.24, 1.81) | <0.001 |
| 2 | 1.38 (1.13, 1.67) | 0.001 | 1.35 (1.11, 1.65) | 0.003 |
| 3 | 1.44 (1.17, 1.77) | 0.001 | 1.39 (1.13, 1.72) | 0.002 |
| 4 | 1.54 (1.21, 1.95) | <0.001 | 1.46 (1.15, 1.86) | 0.002 |
| 5 | 1.97 (1.45, 2.69) | <0.001 | 1.84 (1.35, 2.51) | <0.001 |
| Individual MetS components |  |  |  |  |
| Elevated waist circumference | 0.98 (0.88, 1.09) | 0.748 | 0.95 (0.86, 1.06) | 0.402 |
| Elevated HbA1c | 1.22 (1.08, 1.38) | 0.001 | 1.18 (1.04, 1.33) | 0.009 |
| Elevated blood pressure | 1.43 (1.26, 1.62) | <0.001 | 1.44 (1.27, 1.63) | <0.001 |
| Elevated triglycerides | 1.02 (0.92, 1.13) | 0.69 | 1.00 (0.90, 1.11) | 0.962 |
| Reduced HDL cholesterol | 1.05 (0.92, 1.19) | 0.461 | 1.01 (0.89, 1.15) | 0.878 |

The base model adjusted for sociodemographic factors (age, age^2^, sex, ethnicity, and index of multiple deprivation), and the final model adjusted for both socio-demographic factors, and lifestyle factors (smoking status, alcohol drinker status, physical activity, and sleep duration). HR: hazard ratio; CI: confidence interval; IMD: index of multiple deprivation; HbA1c: glycosylated hemoglobin A1c; HDL: high-density lipoprotein; MetS: Metabolic syndrome.

**Table S9. Sensitivity analyses of the association between MetS and incident all-cause stroke.**

| **Sensitivity analysis** | **Base model** | | **Final model** | |
| --- | --- | --- | --- | --- |
|  | **HR (95% CI)** | ***P*** | **HR (95% CI)** | ***P*** |
| Excluding participants with follow-up time <3 years | 1.44 (1.36, 1.52) | <0.001 | 1.39 (1.31, 1.47) | <0.001 |
| Excluding participants with pre-existing cardiovascular disease | 1.39 (1.31, 1.47) | <0.001 | 1.38 (1.31, 1.46) | <0.001 |
| Excluding self-reported stroke cases | 1.38 (1.31, 1.46) | <0.001 | 1.34 (1.27, 1.41) | <0.001 |
| Excluding participants with nontraumatic subarachnoid hemorrhage | 1.34 (1.27, 1.41) | <0.001 | 1.40 (1.34, 1.48) | <0.001 |
| Restricting the analysis to White participants | 1.40 (1.34, 1.48) | <0.001 | 1.35 (1.29, 1.42) | <0.001 |
| Complete-case analysis | 1.39 (1.33, 1.45) | <0.001 | 1.34 (1.28, 1.41) | <0.001 |
| Multiple-imputation analysis | 1.39 (1.33, 1.45) | <0.001 | 1.34 (1.29, 1.40) | <0.001 |

The base model was adjusted for sociodemographic factors, including age, age², sex, ethnicity, and index of multiple deprivation. The final model was additionally adjusted for lifestyle factors, including smoking status, alcohol drinking status, physical activity, and sleep duration. MetS, metabolic syndrome; HR, hazard ratio; CI, confidence interval.
